# Autogenous Shrinkage, Microstructure, and Strength of Ultra-High Performance Concrete Incorporating Carbon Nanofibers

**DOI:** 10.3390/ma12020320

**Published:** 2019-01-21

**Authors:** Jacob L. G. Lim, Sudharshan N. Raman, Md. Safiuddin, Muhammad Fauzi Mohd. Zain, Roszilah Hamid

**Affiliations:** 1 Smart and Sustainable Township Research Centre (SUTRA), Faculty of Engineering and Built Environment, Universiti Kebangsaan Malaysia, 43600 UKM Bangi, Malaysia; jacoblimlg@gmail.com (J.L.G.L.); fauzizain@ukm.edu.my (M.F.M.Z.); roszilah@ukm.edu.my (R.H.); 2 Centre for Innovative Architecture and Built Environment (SErAMBI), Faculty of Engineering and Built Environment, Universiti Kebangsaan Malaysia, 43600 UKM Bangi, Malaysia; 3 Angelo DelZotto School of Construction Management, George Brown College, 146 Kendal Avenue, M5T 2T9 Toronto, Canada; 4 Department of Civil Engineering, Ryerson University, 350 Victoria Street, M5B 2K3 Toronto, Canada

**Keywords:** autogenous shrinkage, carbon nanofibers (CNFs), compressive strength, entrapped air, flowability, microstructure, nanostructure, particle grading, ultra-high performance concrete (UHPC)

## Abstract

The mix design of ultra-high performance concrete (UHPC) is complicated by the presence of many “ingredients.” The fundamental packing density allows a simpler mix design with fewer ingredients to achieve optimum packing density and dense microstructure. The optimum particle grading increases the flowability of UHPC and eliminates entrapped air. This study presents a simplified particle grading design approach that positively influences the strength, autogenous shrinkage, and microstructure characteristics of UHPC. Carbon nanofibers (CNFs) of superior mechanical properties were added to enhance the strength of UHPC and to reduce its autogenous shrinkage. In addition, ground granulated blast-furnace slag (GGBS) was used as a cement replacement material to reduce the amount of cement in UHPC mixes. Test results showed that the presence of homogeneously dispersed CNF increased the compressive strength and compensated the autogenous shrinkage of UHPC. The findings indicated that an ideal particle distribution, which is close to the modified Andreasen and Andersen grading model, contributed to achieving high compressive strength and CNFs were capable of providing nano-bridges to compensate the shrinkage caused by GGBS.

## 1. Introduction

The recent developments in concrete engineering and technology have facilitated researchers around the world to synthesize ultra-high performance concrete (UHPC) with advanced engineering properties. UHPC is characterized by ultra-high strength and durability with or without fiber reinforcement and exhibits a 28-day compressive strength of more than 150 MPa [1,2,3]. However, concrete becomes less ductile when its strength is increased. Therefore, steel fibers or synthetic fibers are commonly used in UHPC to create ultra-high performance fiber-reinforced concrete (UHPFC) with ductile behavior and enhanced mechanical properties. The popular UHPFCs available around the world include Lafarge-patented Ductal, CorTUF by US Army Corps of Engineers, and BSI and Densit in Europe [4,5]. The consistency and quality control of the materials of UHPC are major concerns for its applications. UHPC is widely used for bridges in the United States, where the design of UHPC is generally more complex than that of conventional concrete [6]. The design of UHPC can be formed with eight to ten different ingredients. Due to the complexity of the concrete mix and the involvement of different material parameters, the properties of UHPC vary and it needs to be subjected to special mix design and curing regime. The use of supplementary cementitious materials, such as fly ash (FA), silica fume (SF), and granulated blast-furnace slag (GGBS) to replace part of ordinary Portland cement (OPC) is a common practice to improve the durability of UHPC. FA and GGBS are among the most common supplementary cementitious materials which allow a high percentage of cement replacement. FA at 20–30% cement replacement is able to improve the toughness and compressive strength of concrete [7], while GGBS at 50–70% cement replacement is suitable for mass concreting applications and improving the later age strength [8].

Several particle grading models have been discussed in literature [9,10], and the shape and angularity of every material differ. Lower material variability contributes to achieving a much precise result in terms of optimum grading based on the particle size distribution. Yu et al. [10] proposed the modified Andreasen and Andersen model with a low distribution modulus for the development of UHPFC with a high content of fine particles; the mix design is formulated with cement, limestone powder, quartz powder, and sand. Fennis et al. [11] proposed a model based on packing fraction and grain geometry. In this model, the formulation of mix design includes OPC, GGBS, and FA.

All UHPC designs are based on a low water/cement (w/c) ratio. Theoretically, a low w/c ratio results in high strength. However, UHPC shows an extremely high autogenous shrinkage, thereby leading to cracking at early ages. The early-age cracking due to restrained autogenous shrinkage affects and limits the progress of concrete construction [12]. Low w/c concretes exhibit larger autogenous shrinkage than high w/c concretes. The increasing rate of autogenous shrinkage is also much higher in low w/c concretes. In UHPC including pozzolanic materials, the extent of autogenous shrinkage significantly depends on the water/binder (w/b) ratio and the type of pozzolanic material used in concrete [13,14]. In particular, the autogenous shrinkage strain observed at later ages, that is, 60–90 days, depends on the mix design [13]. Low w/b concretes exhibit large autogenous shrinkage at a faster rate. FA decreases autogenous shrinkage in concrete. In contrast, the inclusion of GGBS increases autogenous shrinkage [8]. The rate and magnitude of autogenous shrinkage for all concretes increase with the increase in curing temperature. However, these effects vary with the w/b ratio and composition of cementitious materials [13]. The use of SF further increases the autogenous shrinkage as a result of its high surface area; the effect is critical in low water content concrete, which thereby undergoes a significantly decreasing internal relative humidity (RH) in cement paste during hardening. In addition, self-desiccation occurs in the absence of an external source of water [12,13].

Nanomaterials such as nano-silica, nano-calcium carbonate, graphite nanoplatelets, carbon nanotubes (CNTs), and carbon nanofibers (CNFs) are used to enhance concrete properties due to their high surface area and fineness [1,15,16]. Both CNTs and CNFs are graphene-based but their morphologies are different. CNFs have a stronger, larger diameter and longer length structure compared to CNTs [16]. CNTs/CNFs are potential candidates in enhancing the characteristics of the nano-scale matrix. CNTs were first introduced by Iijima in 1991 and subsequently adopted in various industries to complement and improve the characteristics of different materials [17]. CNTs/CNFs are nano-dimensional structural ingredients that exhibit extraordinary mechanical properties in terms of Young’s modulus, tensile strength, and flexural capacity. They are potential candidates as nano-reinforcement in concrete due to their nano-dimensional nature and good coverage ability in the cement matrix [16]. The nano-reinforcing characteristics of CNFs facilitate the enhancement in the mechanical properties through filling the nano-pores in concrete, bridging the microcracks formed during loading, and increasing the resistance of concrete’s matrix to crack propagation [18,19]. However, strong Van der Waals forces limit the contribution of CNTs/CNFs in the region where the bonding of these materials with the cement matrix is weak [19,20]. CNTs and CNFs are also challenged by their dispersion issue, which is usually addressed by using a surfactant [18,21]. However, the use of surfactant reduces the bonding strength between cement matrix and CNTs/CNFs; a good dispersion can lead to a positive effect on concrete strength, whereas a poor dispersion can adversely affect the strength of concrete [1,21].

Shimoda et al. [22] reported that the increased content of CNFs resulted in a decrease of bending or flexural strength due to the agglomeration effect of nano-particles [22]. The dispersion of CNFs greatly influences the concrete properties; the micrographs obtained from Scanning Electron Microscope (SEM) have revealed that the presence of individual nanofibers with a good dispersion in the matrix leads to better mechanical properties [23]. Well-dispersed CNFs prolong the post-tension cracking under bending [15,24]. Therefore, a good dispersion of CNFs is essential for higher compressive and flexural strength characteristics in UHPC. Furthermore, autogenous shrinkage is an unavoidable volume reduction in low w/c concretes [25], such as UHPC. Kim et al. [26] found that the addition of CNTs and carbon fibers contributed to a reduction in the autogenous shrinkage. Having said that, there has been very limited information in the literature on the effect of CNTs/CNFs on the autogenous shrinkage behavior of UHPC, which justifies a detailed study in this area.

The present study explores the simplified version of UHPC by optimizing the packing density in the matrix of concrete. GGBS was used as a pozzolanic supplementary cementitious material to increase the strength of concrete. After achieving the two best particle packing options, a new type of CNFs was incorporated to investigate the strength and shrinkage properties of the designated UHPCs under normal curing temperature. The microstructure of hardened UHPCs was also observed by means of a SEM.

## 2. Experimental Investigation

### 2.1. Constituent Materials and Concrete Mixes

**Figure 1:**
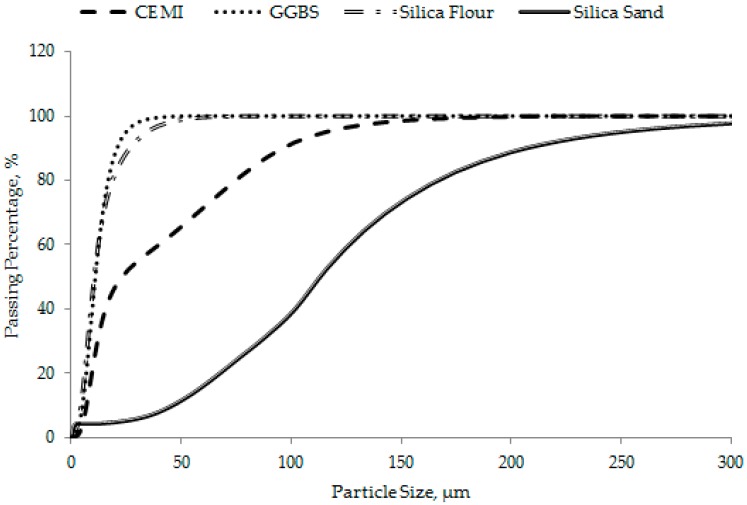
Particle size distribution of different ingredients used in different concrete mixes.

OPC (CEM I 52.5N), silica flour, silica sand, GGBS, superplasticizer (SP), and CNFs were used as the concrete constituent materials. A polycarboxylate-based SP with 35% solid content by weight was used as the water reducing and workability enhancing admixture. The chemical compositions of cement, GGBS, silica flour, and silica sand were determined using the X-ray Diffraction (XRD) technique (D8 Advance, Bruker, Billerica, MA, USA). The results are summarized in Table 1, along with their specific gravity and specific surface area. The silica flour and silica sand contained more than 98% of SiO_2_. The grading (particle size distribution) curves of cement, GGBS, silica flour, and silica sand are shown in Figure 1.

**Figure 2:**
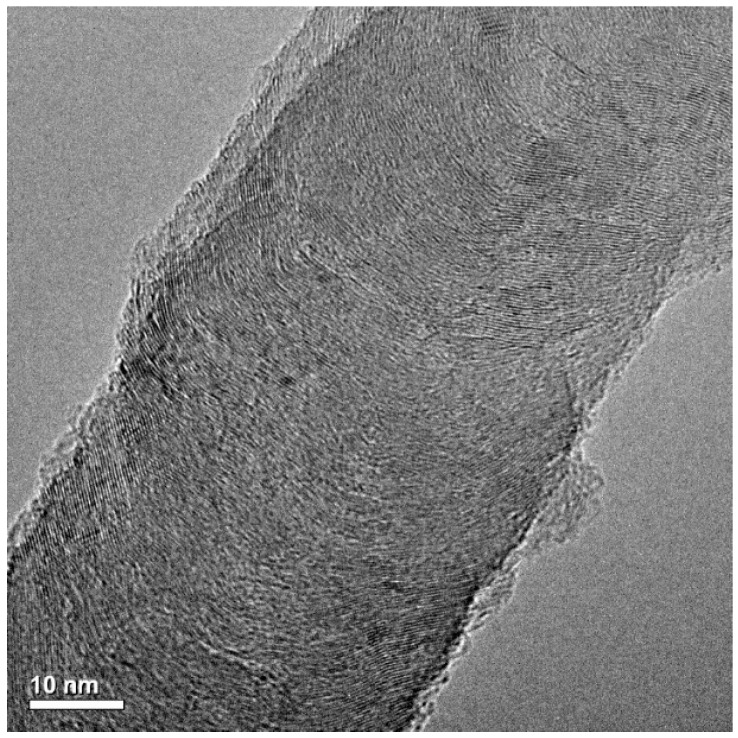
Transmission electron microscope (TEM) image of carbon nanofiber (CNF), a closer nano-structure of an individual fiber.

**Figure 3:**
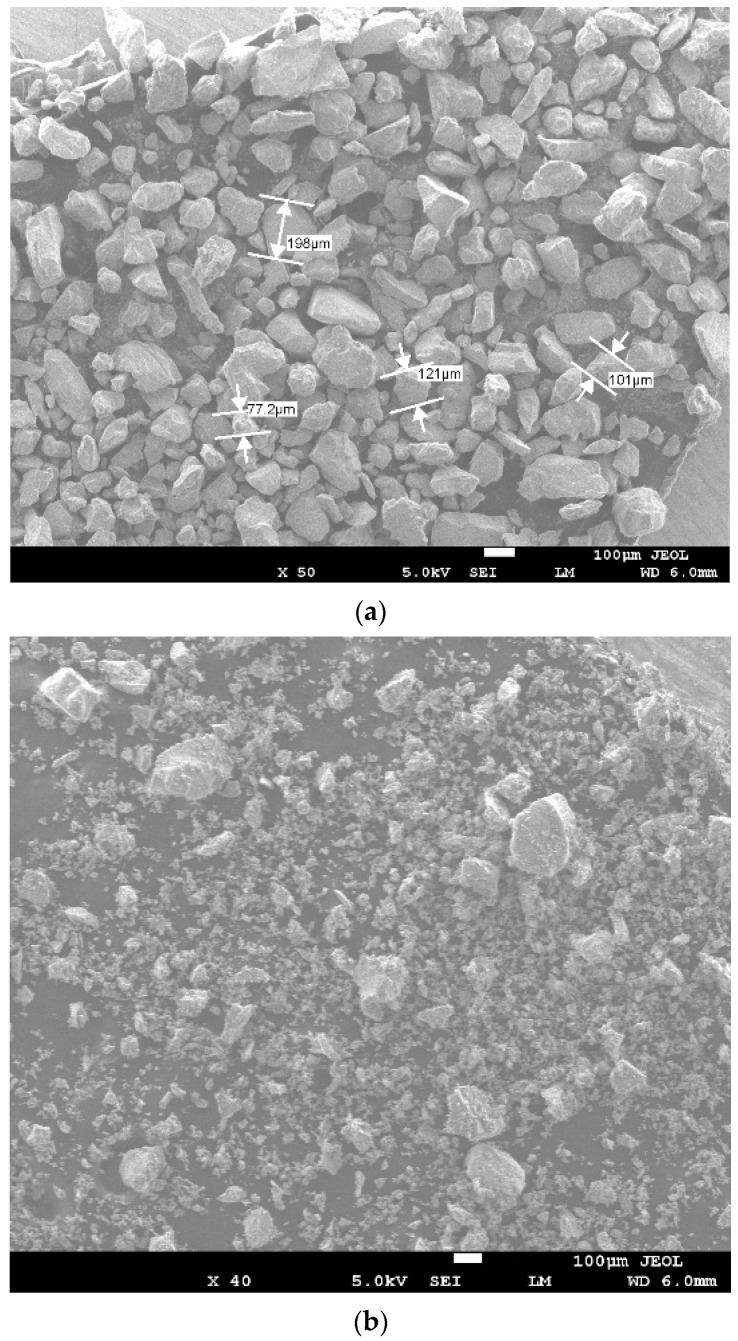
(**a**) Particle morphology of silica sand, (**b**) particle morphology of silica flour.

The selected new type of CNFs with large diameter and length was produced using catalytic chemical vapor deposition technique. Due to a controlled chemical vapor deposition process, the resulting CNFs consisting of the nano-structural filaments in a herringbone structure have stronger and more stable characteristics [27]. Such structure of CNFs, as observed through a transmission electron microscope (TEM, JEOL 2010, Tokyo, Japan), is shown in Figure 2. The selected CNFs had the diameters of 50–250 nm and a length range of 5–10 µm. Before using in concrete, CNFs were dispersed in distilled water through ultrasonication without surface modification and surfactant. The mix proportions of different UHPCs are presented in Table 2. This table shows that silica sand and silica flour were used to produce a number of concrete mixes. The morphology of silica sand is shown in Figure 3a; it was much coarser and flaky in shape. In contrast, silica flour was much finer and spherical in shape; its morphology is shown in Figure 3b. Both silica sand and silica flour were used in producing UHPC mixes because they together provide a better filler effect and improve the microstructure of concrete.

### 2.2. Flow Table Test

**Figure 4:**
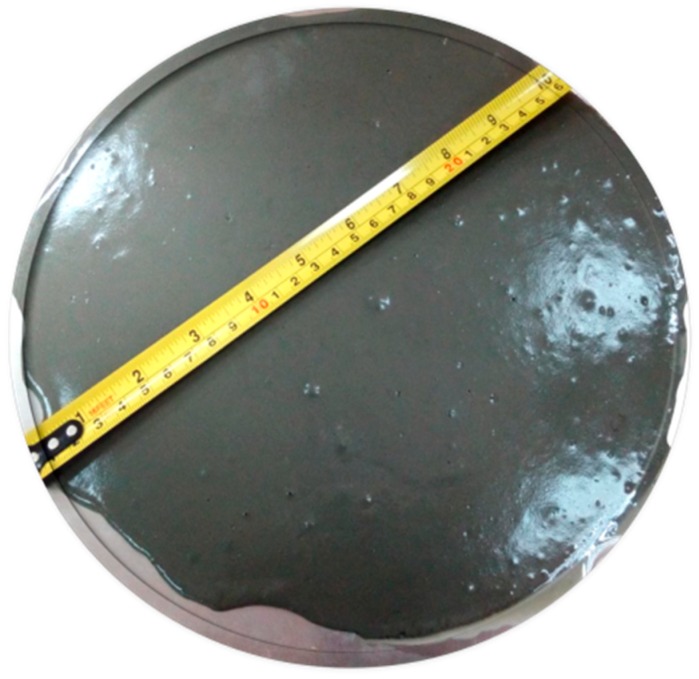
Flow test of concrete Mix 1.

After mixing, the flow table test was performed in accordance with ASTM C230 [28] to evaluate the workability of UHPC with respect to its flowability. The flow was measured for all concrete mixes and the test conducted for Mix 1 is shown in Figure 4.

### 2.3. Compression Test

After conducting the flowability test, the fresh concretes were cast in 50 mm × 50 mm × 50 mm cube molds. The concrete cubes were demolded approximately 24 h after casting and then cured in water at approximately 23 °C. The cured cube specimens were tested at the ages of 1 day, 7 days, and 28 days to determine compressive strength in accordance with ASTM C109 [29]. The results reported herein are the average of three specimens.

### 2.4. Optimization of Particle Packing

**Figure 5:**
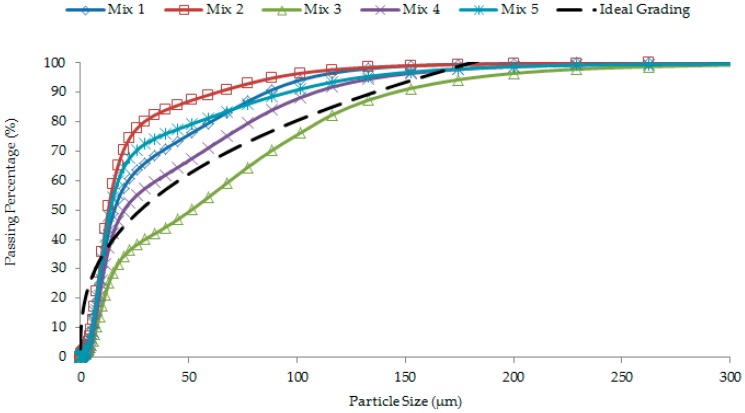
Optimum particle grading of the designated concrete mixes.

Over the years, many researchers studied the best packing of cement or binder (cement plus GGBS) and aggregates through optimum particle grading. In the present study, the cumulative mass of cement or binder and graded sand (silica flour plus silica sand) was calculated based on the modified Andreasen’s curve equation to achieve optimum packing. The combined grading curves of cement or binder and graded sand particles associated with optimum packing for different concrete mixes have been presented in Figure 5.

Different types of concrete can be designed using different values of the distribution modulus, *q*. The optimum design of a high content of fine materials is required in UHPC [10,30]; the value of *q* was fixed at 0.23 in the present study. In the UHPC mix design, the modified Andreasen and Andersen model (Equation (1)) plays the major role to optimize the composition of granular materials. In the study presented herein, all the concrete mixes were designed based on the modified Andreasen and Andersen model as follows:

(1) $$P\left(D\right)=\frac{D^{q}-\;D^{q}min}{D^{q}max-\;D^{q}min}$$

where *D* is the particle size (μm), *P*(*D*) is the fraction of the total solids smaller than size *D*, *Dmax* is the maximum particle size (μm), *Dmin* is the minimum particle size (μm), and *q* is the distribution modulus.

### 2.5. One-Dimensional Autogenous Shrinkage Test

One-dimensional (1D) autogenous shrinkage was measured on the sealed specimens using a stainless steel apparatus by controlling the laboratory temperature at 23 °C and relative humidity at 20%. The specimen’s dimensions were 60 mm in height, 100 mm in width, and 1000 mm in length. External drying was prevented by sealing the specimens immediately after casting using two layers of polyethylene sheets. The free end of the specimen had a linear variable displacement transducer attached to measure the autogenous deformation continuously. The data were recorded every 10 min. The average strain values were reported. This test was performed in accordance with the recommendations given by Wei et al. [11].

### 2.6. Image Analysis for Entrapped Air

The entrapped air content of different concrete mixes was determined based on image analysis. For this test, 40 mm × 40 mm × 5 mm specimens were used. The cross-sections of 1600 mm^2^ for all concrete mixes were obtained from 1-day old 40 mm × 40 mm × 160 mm prism specimens. For each concrete, the prism was cut to a thickness of 5 mm at the center section and oven-dried for 30 min. Then, the prepared 40 mm × 40 mm × 5 mm specimens were placed individually at a fixed object distance of 85 mm in front of the camera lens. The images were captured using a Nikon macro lens (Tokyo, Japan), Nikon 40 mm f/2.8 DX G.

**Figure 6:**
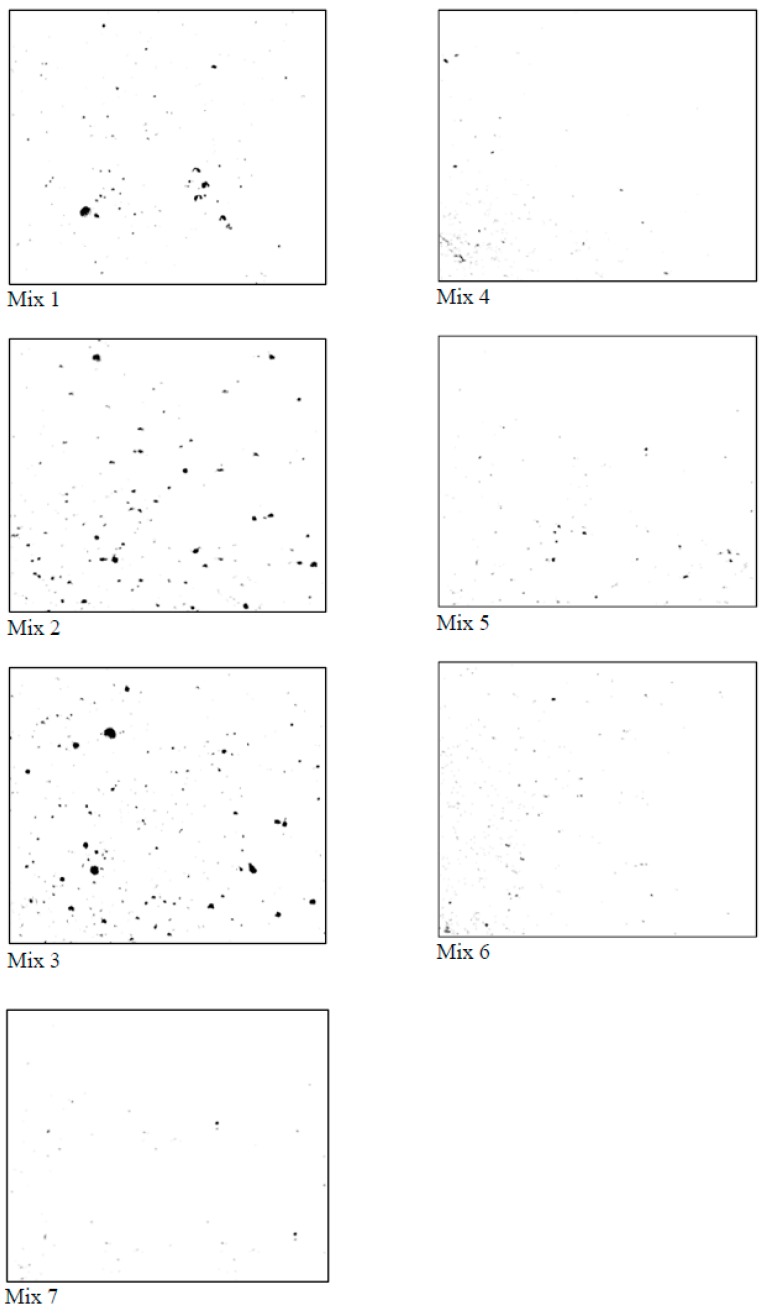
Binary analysis of the cross-section of different concrete mixes.

The cross-sections of all specimens were analyzed using image enhancing and analysis software, ImageJ. All images were set as 8-bit and analyzed using binary (grayscale) analysis, which manually adjusts the threshold level to determine the optimum threshold range to differentiate the surface and depth. In this analysis, the white background represents the concrete surface, whereas the dark background represents the air voids on the surface [31]. The sum of the dark portion represents the entrapped air content, which is calculated as a percentage of the total area. The cross-sectional images of Mix 1 to Mix 7 are shown in Figure 6.

### 2.7. Microstructural/Nanostructural Analysis

The microstructural/nanostructural characteristics of the hardened UHPC specimens were analyzed through the SEM technique, using the Quanta 200 SEM (Field Electron and Ion Company, Hillsboro, OR, USA). For each case, three small-size specimens were prepared from the broken cube specimens, which were tested in compression at 28 days.

The small specimens obtained from broken cubes were dried in an oven, subsequently ground and polished to ensure flattened surface, and then further dried in a desiccator for 24 h before analyzing by the SEM. The dried specimens were coated with gold and pasted with a carbon tape prior to capturing their images. The SEM images were produced using the prepared specimens and analyzed.

## 3. Results and Discussion

### 3.1. Flowability of UHPC

**Figure 7:**
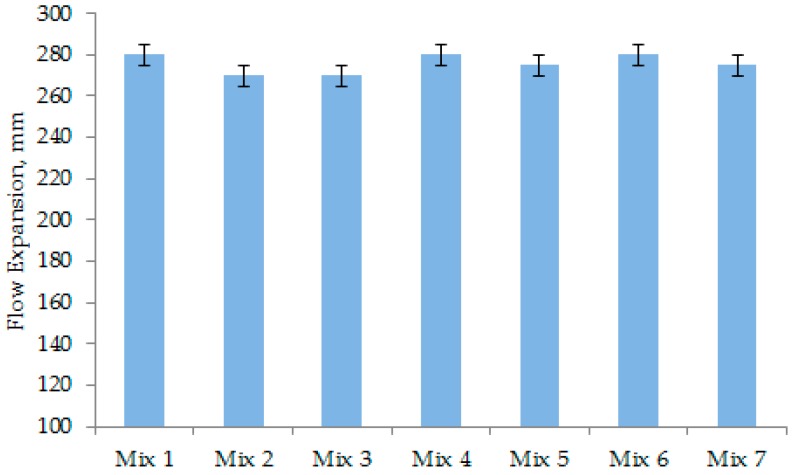
Flowability of the designated concrete mixes.

Mix 1 to Mix 5 with different particle grading characteristics were prepared as the fundamental concrete mixes (without CNFs) to investigate the packing of each mix. The flowability or flow values of all fresh mixes, including Mix 1 to Mix 5, are shown in Figure 7. The flowability reflects the workability, which can predict the denseness in the internal cross-section of a hardened concrete. The target flow value was 280 mm, which should be the ideal flowability to eliminate the entrapped air and achieve good workability with a dense packing. The incorporation of CNFs in Mix 6 and Mix 7 insignificantly affected the flow of those two concrete mixes, as evident from Figure 7.

### 3.2. Entrapped Air of UHPC

**Figure 8:**
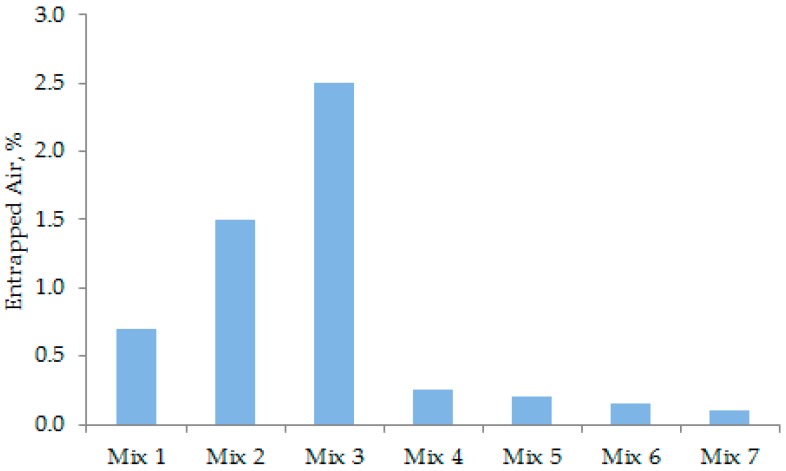
Entrapped air content of different concrete mixes.

The entrapped air contents of different concrete mixes are shown in Figure 8. The amount of entrapped air depended on the flowability of concrete. The large flow can easily eliminate the entrapped air, thus, densifying the concrete mix. Mix 4 and Mix 5 (Figure 6) show a much denser cross-sectional image than Mix 2 and Mix 3. This is because they have significantly less entrapped air, as obvious from Figure 8. The inclusion of CNFs in Mix 6 and Mix 7 did not cause an increase in the entrapped air content of concrete. This is because they had comparable flowability, as evident from Figure 7.

### 3.3. Microstructure/Nanostructure of UHPC

**Figure 9:**
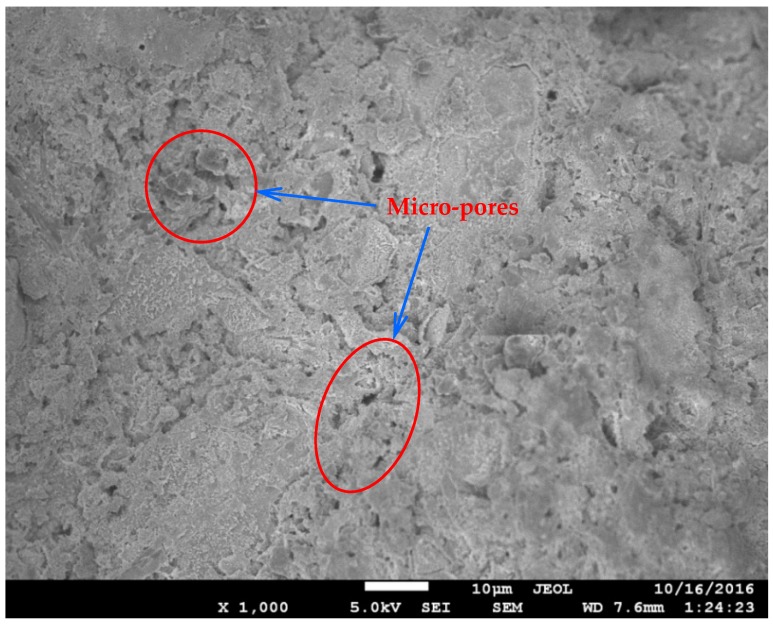
Microstructural image of concrete Mix 4.

**Figure 10:**
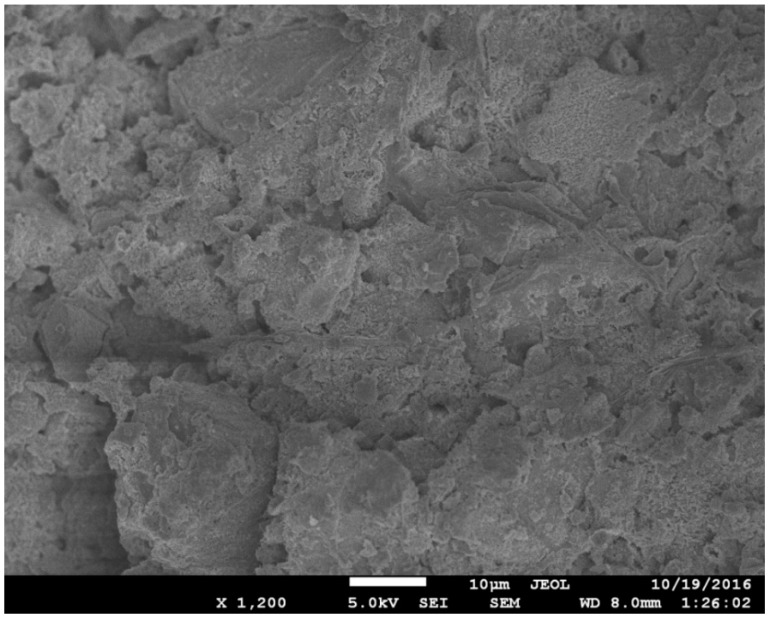
Microstructural image of concrete Mix 5.

**Figure 11:**
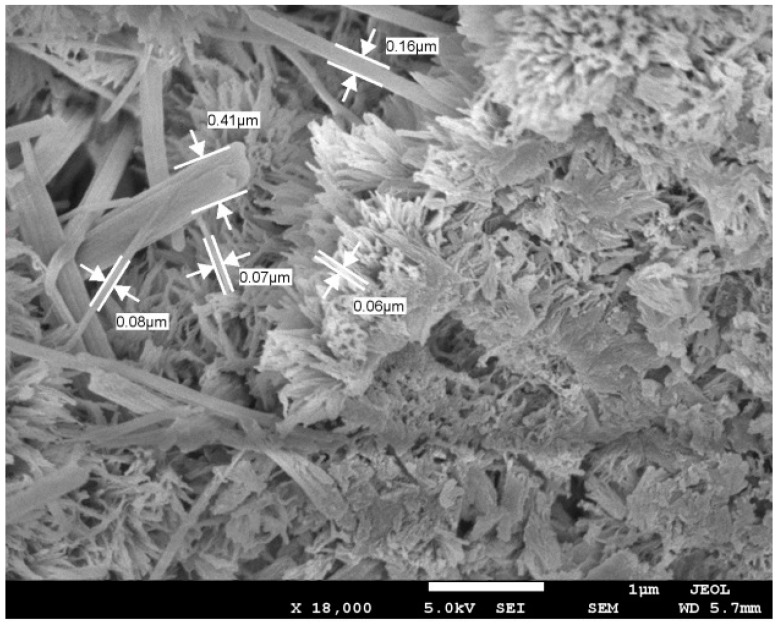
Microstructural image of concrete Mix 7.

The microstructural analysis of different concrete mixes was performed through SEM. In the SEM images, the effects of GGBS and CNFs were particularly observed. Some selected SEM images are given in Figure 9, Figure 10 and Figure 11. Concrete Mix 5, which incorporated GGBS as a 50% replacement of cement, is denser and has a more compacted microstructure than concrete Mix 4, which included OPC as the sole cementitious material (refer to Figure 9 and Figure 10). In Mix 4, the hydrates of OPC have large micro-pores (Figure 9). In Mix 5, the GGBS hydration product filled the micro-pores, thus, making the cement paste much denser (Figure 10). With the addition of CNFs to Mix 5, the microstructure was enhanced due to the nano-reinforcement effect of well-dispersed CNFs and thus a stronger matrix was created. Figure 11 shows the presence of well-bonded and well-dispersed CNFs within the matrix of an UHPC.

### 3.4. Strength Development of UHPC

**Figure 12:**
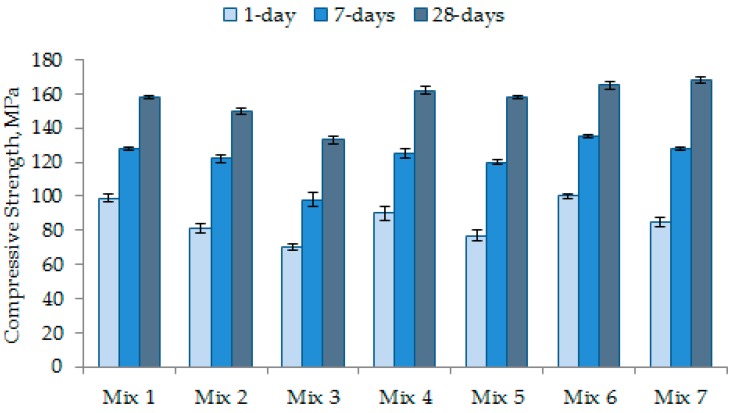
Compressive strength of different concrete mixes.

The compressive strength values of all concrete mixes at specific ages are shown in Figure 12. Mix 1 to Mix 5 were designed to reveal the effects of particle grading. Mix 6 and Mix 7 were particularly designed to examine the effects of CNFs in addition to particle grading. Figure 12 shows that Mix 2 and Mix 3 had lower strength. Specifically, the 28-day strength of these two concrete mixes is approximately 150 and 133 MPa, respectively. Mix 2 and Mix 3 possessed a lower flow of 260–270 mm (Figure 7), which resulted in relatively high entrapped air content (Figure 8). Theoretically, the entrapped air significantly decreases the compressive strength of concrete because cracks can easily propagate through the weak matrix [1,32]. Among Mix 1–Mix 5, Mix 4 recorded the highest compressive strength of 162 MPa at the age of 28 days (Figure 12). This is because the microstructure of matrix was highly dense in this concrete (Figure 10). Moreover, the grading curves presented in Figure 5 shows that Mix 4 has a particle grading close to the modified Andreasen and Andersen grading model (ideal grading), as this concrete mix obtains a dense cross-section (Figure 6). It suggests that Mix 4 should have the highest level of strength.

With 50% replacement of cement with GGBS, Mix 5 provided a relatively low early strength. However, its 28-day strength was close to that of Mix 4 as evident from Figure 12. The reason is that the pozzolanic reaction of GGBS contributes to the strength development, especially at later ages. The silica and alumina compounds react with calcium hydroxide (Ca(OH)_2_, sourced from GGBS as well as cement hydration) to produce secondary calcium silicate hydrates, which play a crucial role in the strength development at later ages. Figure 10 indicates that Mix 5, which had GGBS used as a 50% replacement of cement, is much denser and has a more compacted microstructure than Mix 4, which has OPC hydrates with large micro-pores (Figure 9).

The incorporation of CNFs did not produce any adverse effect on the compressive strength development in concrete. Mix 6 and Mix 7 had a higher early strength (1-day strength and 7-day strength) than their parent control mixes (Mix 4 and Mix 5, respectively), as can be seen from Figure 12. The 28-day strength of these concrete mixes (Mix 6 and Mix 7) was also slightly higher than that of the control mixes (Figure 12). CNFs improved the microstructure of the matrix significantly in both Mix 6 and Mix 7. The high surface area and coverage (distribution) of CNFs strengthened the overall matrix. To further understand the contribution of CNFs in the matrix, SEM analysis was performed for Mix 7 (Figure 11). CNFs with a different range of diameters perfectly bonded with the matrix of concrete, as revealed in Figure 11. In the presence of CNFs in Mix 6 and Mix 7, the 1-day and 7-day strength values became much higher than those of their parent concrete mixes. Mix 6 provided the highest 1-day and 7-day strength values of 100 and 135 MPa, respectively. The increase in early strength occurred because the nano-particles of CNFs accelerated the early hydration process [33,34] acting as nucleation sites. In addition, the well dispersion of CNFs provides a good coverage and, thus, the nanofibers functionalize effectively in the cement matrix. Hence, the calcium silicate hydrates reinforced with nano-fibers become stronger and result in high compressive strength [35].

The compression test results revealed that the compressive strength of the UHPC mixes is correlated with their flow (Figure 7) and entrapped air content (Figure 8). A greater flow or flowability is desirable to eliminate the entrapped air from the concrete mixes; a lower entrapped air content is likely to increase the compressive strength of UHPC. In this context, it can be seen from Figure 8 that Mix 7 had the lowest entrapped air content and, thus, the highest level of 28-day compressive strength was achieved for this concrete, as evident from Figure 12.

### 3.5. Autogenous Shrinkage of UHPC

**Figure 13:**
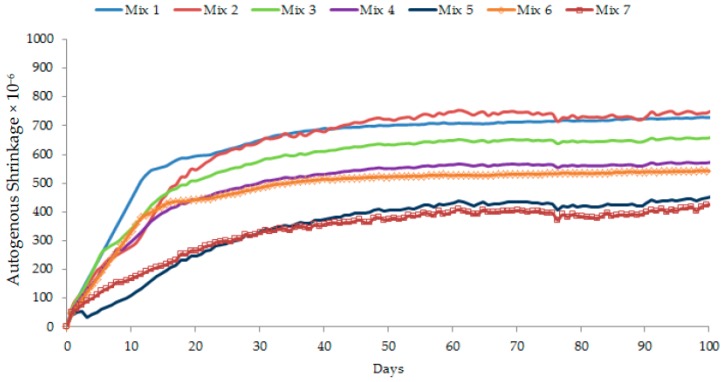
Time-dependent autogenous shrinkage of different concretes.

The age-dependent autogenous shrinkage results for different concrete mixes are shown in Figure 13. Low w/c concrete mixes generally encounter significant autogenous shrinkage owing to considerable self-desiccation during the first several days after casting [12,13]. In the present study, between Mix 1 and Mix 3 with an equal amount of silica particles (the former included finer silica flour whereas the latter incorporated coarser silica sand), Mix 1 with a high specific surface area due to silica flour experienced greater early and ultimate autogenous shrinkage. Mix 3 formed with coarse particle packing due to silica sand experienced lower shrinkage. A refined pore-structure due to finer particle grading caused greater stress on the pore walls and, therefore, generated a higher shrinkage in the case of Mix 1. However, Mix 4 had lower autogenous shrinkage than Mix 3 (Figure 13) even though the former mix possessed a finer particle grading than the latter mix (Figure 5). This is because the particle grading of Mix 4 was more identical to the ideal particle grading than Mix 3. It means that Mix 4 would have the lowest total porosity, which can be justified from its highest level of compressive strength among Mix 1–Mix 5 (Figure 12). Therefore, Mix 4 exhibited lower autogenous shrinkage than Mix 3.

In the present study, Mix 2 and Mix 5 were prepared using GGBS as a 50% replacement of cement. The use of GGBS decreased the early-age autogenous shrinkage but increased the long-term shrinkage for Mix 2. In the case of Mix 5, both the early-age and long-term shrinkage were relatively low. This is probably because Mix 5 contained a lower amount of silica flour than Mix 2. The early-age shrinkage was also reduced because the pozzolanic reaction of GGBS is initially slower than the hydration of cement. When GGBS behaves similar to a filler (that is, prior to the onset of pozzolanic reaction), the increased GGBS content in total cementitious material also increases the effective w/c ratio due to a lower amount of cement. Accordingly, the early-age autogenous shrinkage is decreased. Also, for a lower amount of cement, the available water content for hydration becomes relatively high. This implies that self-desiccation as a result of the lack of water supply cannot develop over time [13,14]. Therefore, the autogenous shrinkage was significantly lower in the case of Mix 5.

The incorporation of CNFs contributed to decreasing the autogenous shrinkage in UHPC. In Mix 6 and Mix 7, CNFs dispersed homogeneously in water beneficially provided sufficient water during hydration. CNFs at a good dispersion stage also impart uniform distribution of nanofibers in the matrix of concrete and, thus, bridge fine cracks and reduce autogenous shrinkage [25,33]. In the present study, CNFs compensated the later-age (after 20–30 days) shrinkage or ultimate shrinkage. This is obvious from the comparison of the shrinkage values of Mix 6 and Mix 7 with those of Mix 4 and Mix 5 (Figure 13). In the absence of CNFs, Mix 4 and Mix 5 had higher ultimate shrinkage mainly due to silica flour. The incorporation of CNFs and their proper dispersion decreased the shrinkage effect of silica flour. This observation is proven by the significant coverage of CNFs in Mix 7 (Figure 11). The well dispersion of CNFs without using any surfactant gave a much stronger bonding between nano-fibers and cement matrix. The natural formation of CNFs with different diameters and lengths further enhanced the nano-bridging between capillary pores and nano- or micro-pores (Figure 11). In such cases, CNFs acted as restraints to limit the shrinkage in concrete.

## 4. Conclusions

The present study demonstrated that optimum particle grading is desirable for dense microstructure and high compressive strength. As a 50% replacement of cement, GGBS addition mainly contributed to providing high strength at the later ages of concrete owing to the pozzolanic reaction. The use of CNFs contributed to decreasing the later-age autogenous shrinkage of UHPC. From this study, the following conclusions are drawn:

1. The flow or flowability of concrete mix directly indicates its entrapped air content. A higher flowability contributes to reducing the entrapped air in concrete.
2. The higher entrapped air content decreases the compressive strength as the air-voids act as a weak link in stress transfer.
3. The ideal particle distribution, which is close to the modified Andreasen and Andersen grading greatly contributes to achieving high compressive strength.
4. The fine particle grading increases the autogenous shrinkage in the matrix of concrete due to a greater specific surface area and more fine pores.
5. CNFs provide nano-bridges in fine cracks to compensate the autogenous shrinkage caused by silica flour.
6. CNFs improve the microstructure or nanostructure of the overall matrix in concrete through good dispersion and uniform distribution of the nanofibers.

## Figures and Tables

**Table 1:** Major properties of solid materials used in concrete mixes.

Properties	OPC	GGBS	Silica Sand	Silica Flour
Specific gravity	3.15	2.99	2.60	2.60
Specific surface area (m^2^/kg)	365	410	120	600
SiO_2_ (%)	21.00	39.00	98.7	99.5
Al_2_O_3_ (%)	5.31	12.50	0.3	0.1
Fe_2_O_3_ (%)	3.44	0.30	0.3	0.1
CaO (%)	65.00	39.50	-	-
MgO (%)	1.50	4.10	0.4	0.1
SO_3_ (%)	0.26	-	-	-
Na_2_O (%)	0.50	0.35	-	-
K_2_O (%)	0.25	0.75	0.3	0.1

**Table 2:** Mix proportions of the designated concretes with and without CNFs.

Mix	Cement	GGBS	Silica Flour	Silica Sand	Water	SP	CNFs
1	1.00	-	0.40	-	0.22	0.012	-
2	0.50	0.50	0.40	-	0.22	0.008	-
3	1.00	-	-	0.40	0.22	0.010	-
4	1.00	-	0.25	0.15	0.22	0.012	-
5	0.50	0.50	0.25	0.15	0.22	0.008	-
6	1.00	-	0.25	0.15	0.22	0.011	0.00067
7	0.50	0.50	0.25	0.15	0.22	0.008	0.00067

## References

[1] Lim J.L.G., Raman S.N., Hamid R., Zain M.F.M., Lai F.C. Synthesis of ultra-high performance cementitious composite incorporating carbon nanofibers. Proceedings of the 6th International Conference on Structural Engineering and Construction Management.

[2] Wille K., Naaman A.E., Parra-Montesinos G.J. (2011). Ultra-high performance concrete with compressive strength exceeding 150 MPa (22 ksi): A simpler way. ACI Mater. J..

[3] Raman S.N., Mohamed M.F., Raman S.N. (2012). New generation concrete in construction: 150 MPa and beyond. Between: Form + Being.

[4] Prem P.R., Bharatkumar B.H., Iyer N.R. (2012). Mechanical properties of ultra-high performance concrete. Int. J. Civ. Environ. Struct. Constr. Arch. Eng..

[5] Acker P., Behloul M. Ductal® technology: A large spectrum of properties, a wide range of applications. Proceedings of the International Symposium on Ultra High Performance Concrete.

[6] Graybeal B., Davis M. (2008). Cylinder or cube: Strength testing of 80 to 200 MPa (116 to 29 ksi) ultra-high-performance-fiber-reinforced concrete. ACI Mater. J..

[7] Golewski G.L. (2017). Generalized fracture toughness and compressive strength of sustainable concrete including low calcium fly ash. Materials.

[8] Tazawa E., Miyazawa S. (1995). Influence of cement and admixture on autogenous shrinkage of cement paste. Cem. Concr. Res..

[9] Brouwers H.J.H., Radix H.J. (2005). Self-compacting concrete: Theoretical and experimental study. Cem. Concr. Res..

[10] Yu R., Spiesz P., Brouwers H.J.H. (2014). Effect of nano-silica on the hydration and microstructure development of ultra-high performance concrete (UHPC) with a low binder amount. Constr. Build. Mater..

[11] Fennis S.A.A.M., Walraven J.C., den Uijl J.A. (2009). The use of particle packing models to design ecological concrete. Heron.

[12] Wei Y., Hansen W., Biernacki J.J., Schlangen E. (2011). Unified shrinkage model for concrete from autogenous shrinkage test on paste with and without ground-granulated blast-furnace slag. ACI Mater. J..

[13] Jiang C., Yang Y., Wang Y., Zhou Y., Ma C. (2014). Autogenous shrinkage of high performance concrete containing mineral admixtures under different curing temperatures. Constr. Build. Mater..

[14] Lim S.N., Wee T.H. (2000). Autogenous shrinkage of ground granulated blast furnace slag concrete. ACI Mater. J..

[15] Meng W., Khayat K.H. (2016). Mechanical properties of ultra-high-performance concrete enhanced with graphite nanoplatelets and carbon nanofibers. Compos. B Eng..

[16] Li W.W., Ji W.M., Wang Y.C., Liu Y., Shen R.X., Xing F. (2015). Investigation on the mechanical properties of a cement-based material containing carbon nanotube under drying and freeze-thaw conditions. Materials.

[17] Iijima S. (1991). Helical microtubules of graphitic carbon. Nature.

[18] Lim J.L.G., Raman S.N., Lai F.C., Zain M.F.M., Hamid R. (2017). Synthesis of nano cementitious additives from agricultural wastes for the production of sustainable concrete. J. Clean. Prod..

[19] Siddique R., Mehta A. (2014). Effect of carbon nanofibers on properties of cement mortars. Constr. Build. Mater..

[20] Collins F., Lambert J., Duan W.H. (2012). The influences of admixtures on the dispersion, workability, and strength of carbon nanotubes-OPC paste mixtures. Cem. Concr. Compos..

[21] Chen Z., Lim J.L.G., Yang E.H. (2016). Ultra high performance cement-based composites incorporating low dosage of plasma synthesized carbon nanofibers. Mater. Des..

[22] Shimoda K., Hinoki T., Kohyama A. (2010). Effect of carbon nanofibers (CNFs) content on thermal and mechanical properties of CNFs/SiC nanocomposites. Compos. Sci. Technol..

[23] Ardanuy M., Rodríguez-Perez M.A., Algaba I. (2011). Electrical conductivity and mechanical properties of vapor-grown carbon nanofibers/trifunctional epoxy composites prepared by direct mixing. Compos. Part B.

[24] Wang H., Gao X., Liu J., Ren M., Lu A. (2014). Multi-functional properties of carbon nanofiber reinforced reactive powder concrete. Constr. Build. Mater..

[25] Wu L., Farzadnia N., Shi C., Zhang Z., Wang H. (2017). Autogenous shrinkage of high performance concrete: A review. Constr. Build. Mater..

[26] Kim G.M., Yoon H.N., Lee H.K. (2018). Autogenous shrinkage and electrical characteristics of cement pastes and mortars with carbon nanotube and carbon fiber. Constr. Build. Mater..

[27] Yehia M.M., Ihsanullah A.S., Al-Ansari T., Atieh M.A. (2018). A review of carbon nanomaterials’ synthesis via the chemical vapor deposition (CVD) method. Materials.

[28] ASTM International (2014). ASTM C230/C230M-14. Standard Specification for Flow Table for Use in Tests of Hydraulic Cement.

[29] ASTM International (2016). ASTM C109/C109M-16. Standard Test Method for Compressive Strength of Hydraulic Cement Mortars.

[30] Yu R., Spiesz P., Brouwers H.J.H. (2015). Development of an eco-friendly ultra-high performance concrete (UHPC) with efficient cement and mineral admixtures uses. Cem. Concr. Compos..

[31] Chini A.R., Villavicencio E.J. Detection of microcracks in concrete cured at elevated temperature. Proceedings of the International Conference on Recent Advances in Concrete Technology.

[32] Mindess S., Young J.F., Darwin D. (2003). Concrete.

[33] Yazdanbakhsh Z., Grasley B., Tyson R., Al-Rub K.A. (2010). Distribution of carbon nanofibers and nanotubes in cementitious composites. Transp. Res. Rec..

[34] Makar J.M., Chan G.W. (2009). Growth of cement hydration products on single walled carbon nanotubes. J. Am. Ceram. Soc..

[35] Nasibulin A.G., Shandakov S.D., Nasibulina L.I., Cwirzen A., Mudimela P.R., Cwirzen K.H. (2009). A novel cement-based hybrid material. New J. Phys..

